# Morphine Modulates Interleukin-4- or Breast Cancer Cell-induced Pro-metastatic Activation of Macrophages

**DOI:** 10.1038/srep11389

**Published:** 2015-06-16

**Authors:** Samira Khabbazi, Yannick Goumon, Marie-Odile Parat

**Affiliations:** 1 University of Queensland School of Pharmacy, PACE, 20 Cornwall Street, Woollloongabba QLD 4102, Australia; 2 CNRS UPR3212, Institut des Neurosciences Cellulaires et Intégratives, Centre National de la Recherche Scientifique and University of Strasbourg, 5 rue Blaise Pascal, 67084 Strasbourg, France

## Abstract

Interactions between cancer cells and stromal cells in the tumour microenvironment play a key role in the control of invasiveness, metastasis and angiogenesis. Macrophages display a range of activation states in specific pathological contexts and alternatively activated (M2) macrophages can promote tumour aggressiveness. Opioids are able to modulate tumour growth and metastasis. We tested whether morphine modulates the activation of macrophages induced by (i) interleukin-4 (IL-4), the prototypical M2 polarization-inducing cytokine, or (ii) coculture with breast cancer cells. We showed that IL-4 causes increased MMP-9 production and expression of the alternative activation markers arginase-1 and MRC-1. Morphine prevented IL-4-induced increase in MMP-9 in a naloxone- and methylnaltrexone-reversible fashion. Morphine also prevented IL-4-elicited alternative activation of RAW264.7 macrophages. Expression of MMP-9 and arginase-1 were increased when RAW264.7 were subjected to paracrine activation by 4T1 cells, and this effect was prevented by morphine via an opioid receptor-mediated mechanism. Morphine further decreased 4T1 breast cancer cell invasion elicited by co-culture with RAW264.7. Reduction of MMP-9 expression and alternative activation of macrophages by morphine was confirmed using mouse bone marrow-derived macrophages. Taken together, our results indicate that morphine may modulate tumour aggressiveness by regulating macrophage protease production and M2 polarization within the tumour microenvironment.

Opioids display a range of activities that have been proposed to both promote and inhibit tumour growth and metastasis^1^. There is considerable interest in unravelling whether opioids administered in the perioperative period to cancer surgery patients can influence tumour recurrence or metastasis^2^. Opioids are both perceived as a threat in the context of cancer (because of their immune suppressive effects, which may impair defences against cancer cells^3^, their pro-angiogenic potential^4^, and the pro-tumour effects of μ opioid receptor^5^), and as beneficial due to the tumour-promoting effects of pain^6^, and the anti-tumour potential of opioid agonists^7^,^8^. Our work has previously shown that morphine regulates the interaction between breast cancer cells and stromal cells, and more specifically moderates the paracrine action of macrophages on the proteolytic profile of breast cancer cells^8^. This posed the question of whether morphine could also regulate macrophage phenotype and behaviour. The plasticity of macrophages has been the object of recent interest which unveiled their ability to adopt multiple possible phenotypes situated between the two extremes of classical (M1) or alternative (M2) activation. In this somewhat simplified paradigm, the M1 phenotype, characterized by high expression of pro-inflammatory cytokines and high production of reactive oxygen and nitrogen species, has enhanced microbicidal ability and plays an important role in the early phase of inflammation, whereas M2 macrophages promote tissue remodelling and resolution of inflammation^9^,^10^. In cancer, M1 macrophages exhibit anti-tumour activity while tumour progression is associated with, and promoted by, M2 macrophages^9^. Tumour associated macrophages (TAMs) share phenotypical and functional features with M2 macrophages^10^. IL-4-expressing Th2 lymphocytes regulate the phenotype and behaviour of TAMs *in vivo*, resulting in increased breast cancer metastasis^11^. A recent report challenged the fact that TAMs are alternatively activated^12^, suggesting further complexity in macrophage activation.

In the present study, we employed two separate models of *in vitro* activation of macrophages, namely IL-4 exposure and paracrine activation by breast cancer cells. We assessed the effect of morphine on expression of M2 markers and the production of matrix proteases in both experimental models.

## Results

### Alternative activation of RAW264.7 macrophages by IL-4

**Figure 1:**
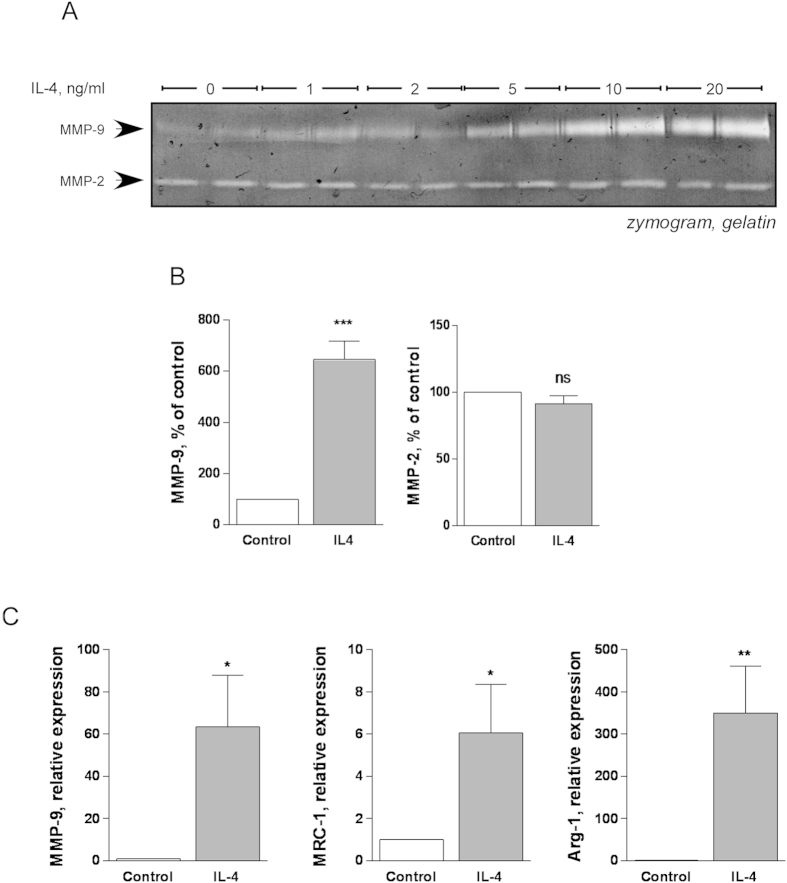
Alternative activation of RAW264.7 macrophages by IL-4. **A**) Production of gelatinases MMP-9 and MMP-2 in the conditioned medium of RAW264.7 cells treated with indicated concentrations of IL-4 for 48 h was assessed by gelatine zymography. **B**) RAW264.7 cells were treated with 5 ng/ml IL-4 for 48 h and gelatinase production determined by zymography followed by densitometric quantitation of MMP-9 and MMP-2. Mean ± SEM is shown, n  = 3 independent experiments. ***p < 0.001; ns, no statistically significant difference, Student’s *t*-test **C**) Real-time RT-PCR determination of mRNA levels of MMP-9 as well as alternative activation markers MRC-1 and arginase-1 (Arg-1) in RAW264.7 cells treated with 5 ng/ml IL-4 for 48h. Results are expressed relative to untreated control. Mean ± SEM is shown, n = 7–10 independent experiments. *p < 0.05, **p < 0.01, Student’s *t*-test.

IL-4 is documented to be the prototypic cytokine that induces alternative activation of macrophages^10^. To establish an *in vitro* model of alternative macrophage activation, we incubated RAW264.7 cells with 1 to 20 ng/ml IL-4 in serum-free medium for 48 h. The conditioned medium was collected and analysed by gelatine zymography (Fig. 1A). At doses of 5, 10 and 20 ng/ml, IL-4 increased the production of MMP-9, while MMP-2 was unaffected. The smallest dose tested that induced a statistically significant increase in MMP-9 was 5 ng/ml (Fig. 1B, p = 0.0008). The increased expression of MMP-9 in IL-4-treated cells was confirmed using real time RT-PCR (Fig. 1C, p = 0.0104). We further confirmed that this concentration of IL-4 (5ng/ml) elicited increased expression of the alternative activation markers MRC-1 and arginase-1 (Fig. 1C). The increase was statistically significant for both markers (p = 0.0244 and 0.0018, respectively). In our study, as the relative increase in expression of arginase-1 was higher than that of MRC-1, we employed arginase-1 mRNA expression to measure alternative activation of RAW264.7 cells in subsequent experiments.

### Morphine prevents IL-4 induced increase in MMP-9 production, and expression of alternative activation markers in RAW264.7 macrophages

**Figure 2:**
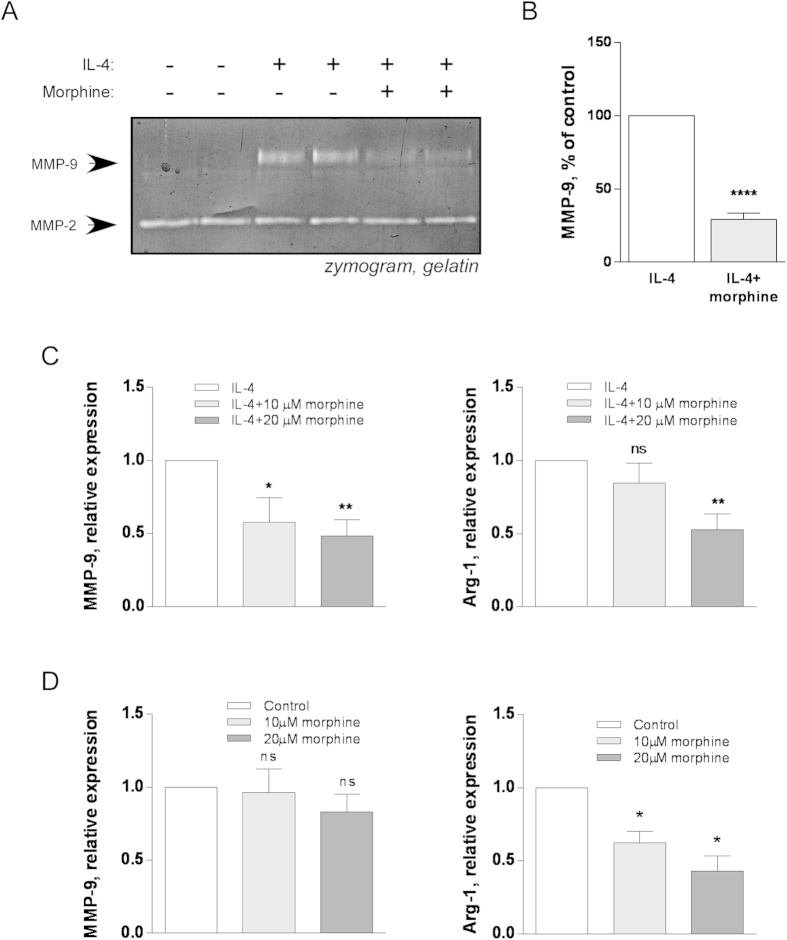
Morphine inhibits IL-4 induced increase of MMP-9 production by RAW264.7 macrophages. **A**) RAW264.7 macrophages were incubated with IL-4 (5 ng/ml) in the presence or absence of morphine (20 μM). Control cells were incubated with buffer alone. Conditioned media were tested for gelatinase production by zymography. **B**) Zymography gels were subjected to densitometric quantitation of MMP-9. Mean ± SEM is shown, n = 3 separate experiments. ****p < 0.0001, Student’s *t*-test. ; **C**) Real-time RT-PCR determination of mRNA levels of MMP-9 and alternative activation marker arginase-1 (Arg-1) or MRC-1 in RAW264.7 cells treated with 5 ng/ml IL-4 and either 0, 10 or 20 μM morphine for 48 h. Results are expressed relative to IL-4 only treated cells. Mean ± SEM is shown, n = 3–6 independent experiments. *p < 0.05; **p < 0.01; ns, no statistical significance, One way ANOVA with Dunnet’s multiple comparison test. **D**) Real-time RT-PCR determination of mRNA levels of MMP-9 or alternative activation marker arginase-1 (Arg-1) in RAW264.7 cells treated with 0, 10 or 20 μM morphine for 48h. Results are expressed relative to untreated control. Mean ± SEM is shown, n = 4 independent experiments. ns, no statistical significance; *p < 0.05, One way ANOVA with Dunnet’s multiple comparison test.

To test whether morphine affected IL-4 induced macrophage M2 polarization, RAW264.7 cells were incubated for 48 h in serum-free medium containing either 5 ng/ml IL-4, or 5 ng/ml IL-4 and 20 μM morphine. Zymography of conditioned media showed that morphine prevented the IL-4 induced increase in MMP9 activity (Fig. 2A) and densitometric quantification showed that the decrease was statistically significant (Fig. 2B). MMP-2, in contrast, was unaffected by IL-4 or morphine (Fig. 2A). The effect of morphine on IL-4-induced MMP-9 was detected at mRNA level and statistically significant (p = 0.0032, one way ANOVA) where both 10 and 20 μM morphine were effective (Fig. 2C). Interestingly, morphine also significantly (p = 0.0043, one way ANOVA) prevented the induction by IL-4 of the M2 marker arginase-1 (Fig. 2C). In the absence of IL-4, morphine did not affect the levels of MMP-9 mRNA (Fig. 2D) but significantly reduced arginase-1 mRNA in RAW264.7 cells (p = 0.0048, one way ANOVA).

### The effect of morphine on IL-4 induced increase in MMP-9 production is reversed by opioid receptor antagonists

**Figure 3:**
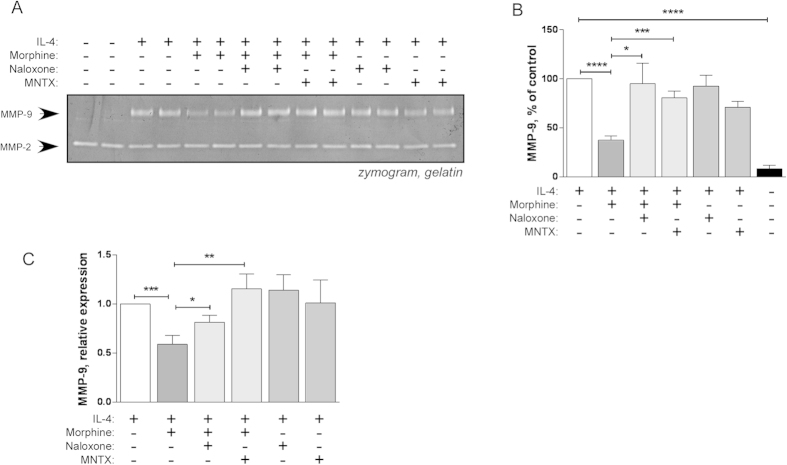
The effect of morphine is opioid receptor-mediated. RAW264.7 macrophages were incubated for 48`h with IL-4 (5 ng/ml) in the presence or absence of morphine (20 μM) and naloxone (20 μM) or methylnaltrexone (MNTX) (20 μM) as indicated. **A**) Conditioned media were collected and analysed by gelatine zymography. **B**) Zymography gels were used for densitometric quantitation. Mean ± SEM is shown, n = 5 independent experiments. *p < 0.05; ***p < 0.001; ****p < 0.0001, Student’s *t* test. **C**) Real-time RT-PCR determination of mRNA levels of MMP-9 in RAW264.7 cells treated as indicated. Results are expressed relative to IL-4 only treated cells. Mean ± SEM is shown, n = 5 independent experiments. *p < 0.05; **p < 0.01; ***p < 0.001, Student’s *t* test.

To test whether the effect of morphine on IL-4 induced macrophage MMP-9 production was opioid receptor-mediated, RAW264.7 cells were incubated for 48 h in serum-free medium containing morphine in the presence or absence of the pure antagonists naloxone or methyl naltrexone at equimolar concentrations (20 μM). Either inhibitor significantly reversed the effect of morphine on MMP-9 activity in the conditioned medium (Fig. 3A,B). MMP-2 was unaffected by all treatments (Fig. 3A). The reversal of morphine’s effect on IL-4-induced increase in MMP-9 by the inhibitors was also evident, and statistically significant, at mRNA level (Fig. 3C). These results indicate that morphine prevents IL-4-induced MMP-9 expression via an opioid receptor-mediated pathway.

### Paracrine activation of RAW264.7 cells by 4T1 breast cancer cells results in MMP-9 induction and alternative activation

**Figure 4:**
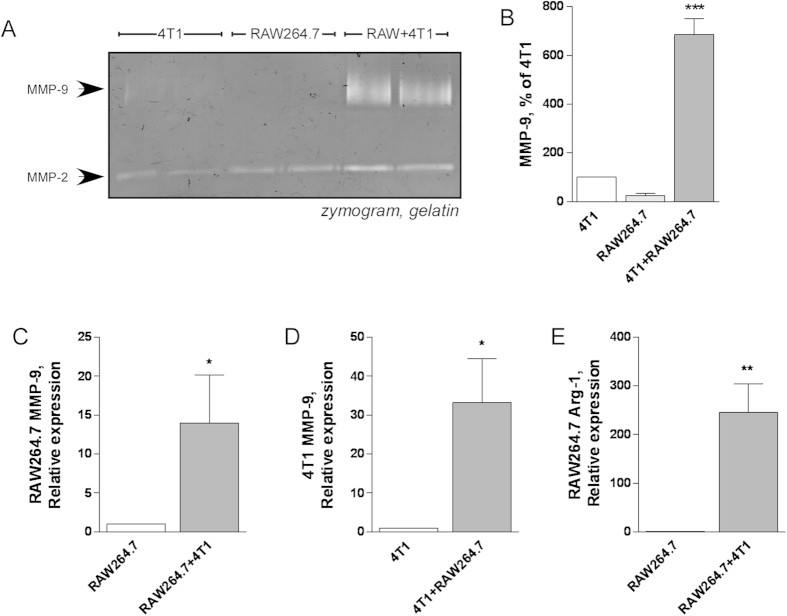
MMP-9 production is increased in Transwell® co-cultures of macrophages and 4T1 cells. RAW264.7 macrophages (in the lower chamber) and 4T1 cells (in the upper chamber) were cultured individually or together for 48 h in 12-well Transwell®. **A**) Conditioned media were collected and production of MMP-9 was determined by gelatine zymography. **B**) Densitometric quantitation of MMP-9 activity in the conditioned media. Mean ± SEM is shown. n = 3 independent experiments ***p < 0.001 compared to 4T1 or RAW264.7 alone, Student’s *t* test. **C-D**) Real-time PCR determination of MMP-9 mRNA levels in RAW264.7 cells (**C**) and 4T1 cells (**D**) alone or co-cultured for 48h. Results are expressed relative to each cell type grown individually. Mean ± SEM is shown, n = 3 independent experiments. *p < 0.05, Student’s *t* test. **E**) Real-time PCR determination of arginase-1 mRNA levels of RAW264.7 cells co-cultured with 4T1 cells for 48 h. Results are expressed relative to RAW264.7 grown individually. Mean ± SEM is shown, n = 3 independent experiments. **p < 0.01, Student’s *t* test.

To test whether increased MMP-9 production and alternative activation of macrophages were achieved by paracrine communication with breast cancer cells, we placed RAW264.7 cells in the presence of 4T1 cells for 48h using Transwells®. Control cells were grown individually. Interaction of the cells resulted in increased production of MMP-9 in the conditioned medium (Fig. 4A,B) compared with the medium of individual cells. To assess which cell type is responsible for increased MMP-9 production, RNA was prepared from each cell type and tested by real time RT-PCR. Results show that both cell types underwent induction of MMP-9 expression when co-cultured (Fig. 4C,D). Lastly, we examined whether RAW264.7 cells underwent alternative activation, and showed that the marker arginase-1 was strongly induced when RAW264.7 cells were exposed to paracrine activation by 4T1 cells (Fig. 4E). This model of M2 activation was next used to test the effect of morphine.

### Morphine modulates breast cancer cell-induced alternative activation of RAW264.7 macrophages

**Figure 5:**
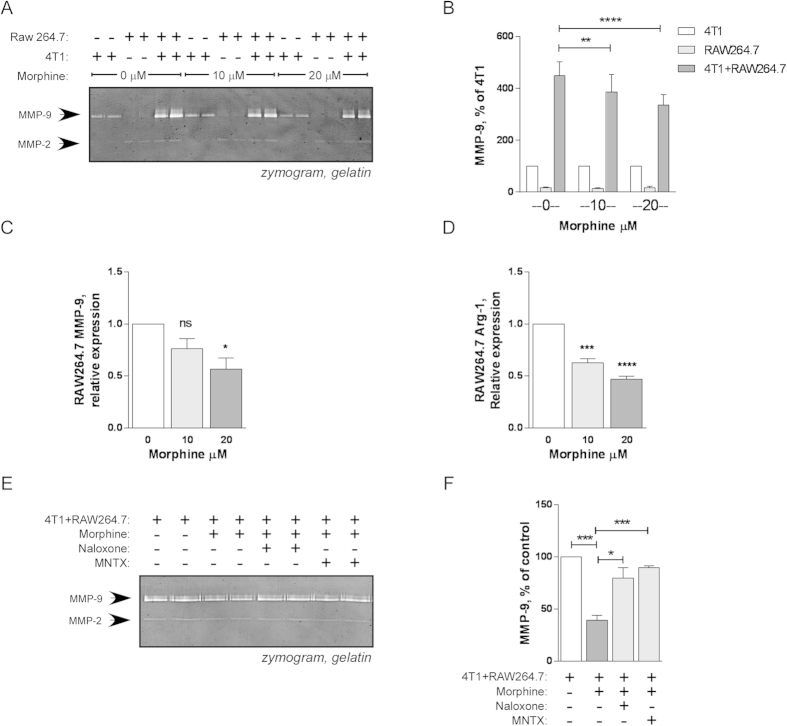
Morphine inhibits coculture-induced increase in MMP-9 production by RAW264.7 macrophages. RAW264.7 macrophages (in the lower chamber) and 4T1 cells (in the upper chamber) were cultured individually or together for 48 h in 12-well Transwell® in the presence (10 or 20 μM as indicated) or absence of morphine. **A**) Production of MMP-9 in the conditioned media was determined by gelatine zymography. **B**) Densitometric quantitation of MMP-9 activity in the conditioned media. Results are expressed as a % of MMP9 in 4T1 alone. Mean ± SEM is shown. n = 3 independent experiments *p < 0.05; ***p < 0.001, Two way ANOVA with Tukey’s multiple comparisons test. **C**) Real-time PCR determination of MMP-9 mRNA levels in RAW264.7 cells co-cultured for 48 h with 4T1 in the presence (10 or 20 μM as indicated) or absence of morphine. Results are expressed relative to RAW264.7 cells co-cultured with 4T1 in the absence of morphine. Mean ± SEM is shown, n = 3 independent experiments. *p < 0.05, One way ANOVA with Dunnet’s multiple comparisons test. **D**) Real-time PCR determination of arginase-1 (Arg-1) mRNA levels in RAW264.7 cells co-cultured for 48 h with 4T1 in the presence (10 or 20 μM as indicated) or absence of morphine. Results are expressed relative to RAW264.7 cells co-cultured with 4T1 in the absence of morphine. Mean ± SEM is shown, n = 3 independent experiments. ***p < 0.001. ****p < 0.0001, One way ANOVA with Dunnet’s multiple comparisons test. **E–F**, RAW264.7 macrophages (in the lower chamber) and 4T1 cells (in the upper chamber) were cultured together for 48 h in 12-well Transwell® in the presence of morphine (20 μM) and naloxone (20 μM) or methylnaltrexone (MNTX) (20 μM) as indicated. **E**) Conditioned media were collected and analysed by gelatine zymography. **F**) Zymography gels were used for densitometric quantitation. Mean ± SEM is shown, n = 3 independent experiments. *p < 0.05; ***p < 0.001, Student’s *t* test.

RAW264.7 and 4T1 cells were grown together or individually for 48 h using Transwells® in the presence of morphine (10 or 20 μM). Control cells were grown together or individually in the absence of morphine. Analysis of the conditioned media by gelatin zymography showed that morphine significantly decreased the coculture-induced surge in MMP-9 production (Fig. 5A,B) (p < 0.01, two way ANOVA) but had no effect on the production of MMP-9 by cells grown individually. The decrease in coculture-induced MMP-9 expression by RAW264.7 was detected at mRNA level (Fig. 5C) and was statistically significant (p = 0.0268, one way ANOVA). Lastly, we tested whether morphine prevented the alternative activation of RAW264.7 induced by coculture with 4T1 cells and showed that the increase in RAW264.7 arginase-1 production was significantly (p < 0.0001, one way ANOVA) reduced by morphine (Fig. 5D).

### The effect of morphine on coculture-induced increase in MMP-9 production is reversed by opioid receptor antagonists

To test whether the effect of morphine on 4T1-induced macrophage MMP-9 production was opioid receptor-mediated, RAW264.7 cells were incubated for 48 h in the presence of 4T1 in Transwells® in serum-free medium containing morphine (20 μM) in the presence or absence of naloxone or methyl naltrexone at equimolar concentrations (20 μM). Either inhibitor significantly reversed the effect of morphine on MMP-9 activity in the conditioned medium (Fig. 5E,F). These results confirm that the effect of morphine on macrophage MMP-9 expression is opioid receptor-mediated.

### Morphine prevents coculture-induced increase in breast cancer cell invasion

**Figure 6:**
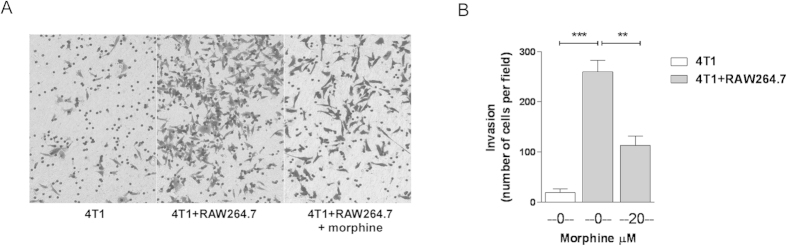
Morphine prevents macrophage-induced breast cancer cell invasion. Invasion of matrigel-coated inserts by 4T1 cells over 48 h was allowed to proceed in the absence or presence of RAW264.7 cells seeded in the bottom well of the chamber, and of 20 μM morphine as indicated. **A**) Representative images of invaded cells after fixation and staining. **B**) Quantification of invaded cells. Mean ± SEM is shown, n = 3 independent experiments. **p < 0.01; ***p < 0.001, Student’s *t* test.

To assess whether the effect of morphine on MMP-9 production had functional consequences, we tested the ability of 4T1 cells to invade basement membrane-like substrate. The cells were seeded in matrigel-coated inserts placed in the absence or presence of RAW264.7 cells in the bottom chamber. In the presence of macrophage cells, breast cancer cell invasion was increased by a factor ~13 (Fig. 6A,B). This was significantly reduced if morphine was added to the co-culture (Fig. 6A,B).

### Morphine prevents IL-4 and coculture-induced increase in MMP-9, Arg-1 and MRC-1 in bone marrow derived mouse macrophages

**Figure 7:**
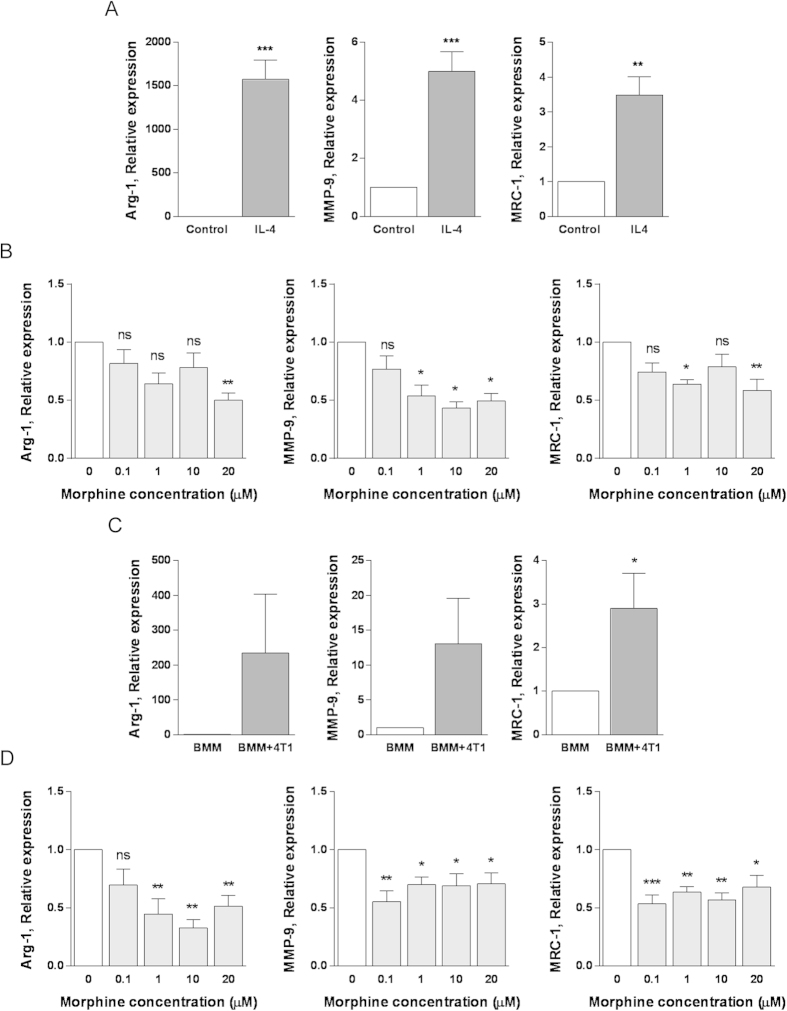
Morphine inhibits alternative activation of bone marrow-derived macrophages (BMM). **A**) BMM were treated with 5 ng/ml IL-4 for 24 h and mRNA expression of MMP-9 and the M2 polarization markers Arg-1 and MRC-1 was assessed by real-time RT-PCR. Results are expressed relative to untreated control. Mean ± SEM is shown, n = 4 independent experiments. **p < 0.01, ***p < 0.001, Student’s *t*-test. **B**) BMM were incubated with IL-4 (5 ng/ml) in the presence or absence of morphine at the indicated concentrations (up to 20 μM) for 24 h and mRNA expression of Arg-1 MMP-9 or MRC-1 was determined by real time RT-PCR. Results are expressed relative to IL-4 only treated cells. Mean ± SEM is shown, n = 3–4 independent experiments. *p < 0.05; **p < 0.01 ns, no statistical significance, One way ANOVA with Dunnet’s multiple comparison test. **C)** BMM and 4T1 cells were cultured individually or together for 24 h in 12-well Transwell® and mRNA levels of Arg-1, MMP-9 and MRC-1 determined by real time RT-PCR in BMM alone or co-cultured with 4T1 cells. Results are expressed relative to BMM grown individually. Mean ± SEM is shown, n = 3–4 independent experiments. *p < 0.05, Student’s *t* test. **D**) BMM were co-cultured with 4T1 in the presence or absence of morphine at the indicated concentrations (up to 20 μM) for 24 h and mRNA expression of Arg-1 MMP-9 or MRC-1 determined by real time RT-PCR. Results are expressed relative to BMM co-cultured with 4T1 in the absence of morphine. Mean ± SEM is shown, n = 3–4 independent experiments. *p < 0.05; **p < 0.01, ***p < 0.001, ns, no statistical significance, One way ANOVA with Dunnet’s multiple comparison test.

To confirm key results obtained using the RAW264.7 cell line, we tested the effect of morphine on IL-4- or 4T1 coculture-induced mRNA expression of MMP-9, Arg-1 and MRC-1. Of note, experiments were performed using 24 h rather than 48 h time points as BMM cannot sustain a 48 h no-serum medium exposure. Both IL-4 (Fig. 7A) and co-culture with 4T1 (Fig. 7C) markedly induced the expression of the three markers, although no statistical significance was reached for Arg-1 and MMP-9 in the case of co-cultures (Fig. 7C). One way ANOVA analysis showed that morphine significantly inhibited the IL-4-induced induction of Arg-1 (p = 0.0296) MMP-9 (p = 0.0225) and MRC-1 (p = 0.0212) (Fig. 7B). Morphine also significantly inhibited the coculture-induced expression of Arg-1 (p = 0.003) MMP-9 (p = 0.0111) and MRC-1 (p = 0.0014) (Fig. 7D). Furthermore the effect of morphine on BMM was observed with much lower doses (starting as low as 100 nM) than in the experiments using RAW264.7 cells.

## Discussion

Our results show a marked increase in MMP-9 expression, as detected by gelatine zymography and real time RT-PCR, when RAW264.7 macrophages are exposed to the M2 polarization-inducing cytokine IL-4 or when they are co-cultured with breast cancer cells in separate chambers. In contrast to MMP-9, there was remarkably no change in MMP-2 in the conditioned medium in either activation model. In the context of tumours, increased MMP-9 is considered to promote invasiveness and angiogenesis, by degradation of the extracellular matrix (ECM) and basement membrane, liberation of matrix-bound growth factors and activation of tumour-promoting agents^13^. Secretion of MMP-9 by tumour-associated macrophages is well documented (reviewed in^14^) and we have previously shown that plating RAW264.7 macrophages and breast cancer cells in contact in coculture altered their proteolytic profile, including increase of MMP-9 production^8^. The link between macrophage exposure to IL-4 and MMP-9 production is less clear, with literature indicating that IL-4 suppresses rather than induces the expression of MMP-9 in macrophages^15^,^16^,^17^,^18^. Discrepancies in MMP production have been attributed to *in vitro* differentiation techniques, plastic or cell-cell contact^19^. In agreement with our data Lolmede *et al.* reported increased MMP-9 production in *ex vivo* polarized human macrophages^20^. Furthermore, we have previously reported that 4T1 tumour-bearing mice exhibit increased circulating MMP-9^8^.

*In vivo*, morphine prevented the increase in circulating MMP-9 elicited by 4T1 tumours^8^. We now show that morphine prevents the increase in MMP-9 elicited by the cross talk between macrophages and breast cancer cells. This corroborates our previous results obtained when cells were in contact in the same dish, or when 4T1 cells were exposed to medium that had been conditioned by RAW264.7^8^. Using Transwells® however, allows reciprocal communication and thus cross talk amplification of signals exchanged between the two cell populations, and the preparation of RNA from each cell type separately to assess their individual contribution to the surge in MMP-9 production. Both 4T1 and RAW264.7 increased transcription of *MMP-9*, in response to reciprocal paracrine activation. Morphine decreased MMP-9 production in the conditioned medium of co-cultured cells without affecting MMP-9 production in cells grown individually. This is in favour of a modulating role for opioids in the tumour microenvironment where they can prevent the matrix degradation (and thus invasion and angiogenesis) resulting from cancer cells’ cross talk with stromal macrophages. The ability of morphine to decrease macrophage-induced invasion of basement membrane like substrate corroborates this. In addition, morphine prevented IL-4-induced increase in MMP-9 production, indicating that opioids may prevent IL-4-expressing Th2 lymphocytes from promoting macrophage activation in the tumour microenvironment.

In their recent study, Doornebal *et al.* quantified and analysed the composition of infiltrating immune cells in mouse breast tumours and showed, using two distinct mouse models, that morphine did not decrease the absolute number of tumour leucocytes or the proportion of infiltrating macrophages^21^. Our finding that morphine qualitatively changes macrophage phenotype in the context of cancer is novel. Morphine decreased the cancer cell coculture- or IL-4-induced expression of arginase-1 in RAW264.7 or bone marrow derived macrophages. This is in agreement with the finding that the endogenous opioid peptide methionine enkephalin (a δ- and to a lesser extent μ-opioid receptor agonist) down-regulates the production of M2 markers CD206 and arginase-1 in tumour associated macrophages *in vivo*^22^. Interestingly, in our experiments while morphine had no effect on MMP-9 production unless the cells were activated by either IL-4 or breast cancer cells, morphine decreased arginase-1 expression in RAW264.7 macrophages in the absence of stimulation. The effect of opioids on macrophage phenotype might therefore not be limited to the tumour microenvironment inflammatory milieu. In a recent report by Godai *et al.* using a tumour-free model, morphine administered locally tilted the balance of macrophages present at a site of incisional inflammation towards the M2 phenotype^23^. Of note, this effect was only seen in the early phase of inflammation (day 2).

The ability of morphine to reduce arginase-1 expression in IL-4- or 4T1 breast cancer cell-activated macrophages is likely to be beneficial in the context of tumours. Arginase activity results in the production of polyamines which promote breast cancer cell proliferation^24^. By competing with nitric oxide synthase (NOS) for arginine as a common substrate, arginase-1 can inhibit nitric oxide (NO) production in LPS-activated macrophages^25^, and NO production is considered to be important for the anti-tumour activity of macrophages. Recently, myeloid cell-specific deletion of arginase-1 in mice was shown to improve residual disease control in a transient window of time after therapy^26^.

In our experiments, morphine prevented MMP-9 increase and arginase-1 induction in both models of activation (treatment with IL-4 and coculture with cancer cells) in RAW264.7 and BMM cells. Together with other cell types, Th2 lymphocytes are believed to be a major source of IL-4 in the tumour microenvironment, leading to activation of macrophages^10^,^11^. We have mimicked this by directly applying IL-4 on RAW264.7 cells *in vitro*. In addition, interaction with breast cancer cells induced MMP-9 and arginase-1. This could be due to IL-4, as cancer cells are also a source of IL-4 capable of eliciting alternative macrophage activation^27^,^28^.

Our results show that both naloxone and methylnaltrexone inhibit the effect of morphine on IL-4- or 4T1 coculture-induced MMP-9 production by macrophages, in favour of an opioid receptor-mediated action. A previous study showing that morphine decreased mRNA expression of MMP-9 in cultured breast cancer cells showed no reversal by equimolar naloxone^29^. Although the experimental design is not comparable to our study because the effect of morphine was not tested in response to a MMP-9-inducing factor but in un-stimulated cells, this is a reminder that morphine exerts both opioid-mediated and -independent effects at cellular level. Our data does not unveil the downstream mechanism by which opioid activation results in modulation of MMP-9, Arg-1 and (in BMM) MRC-1 mRNA expression. We have tested whether the effect of morphine involved STAT-6, a major regulator of IL-4-induced macrophage M2 polarization^9^. Our results (data not shown) confirmed the strong increase in STAT-6 phosphorylation in IL-4-treated RAW264.7 cells, but this was unaffected by morphine, excluding STAT-6 as a mediator of opioid action in our model.

Morphine modulates tumour growth and metastasis via multiple mechanisms, some of which are likely to promote, others likely to prevent, cancer aggressiveness^1^,^30^. The results presented in this study indicate that morphine may attenuate the invasion-promoting effects of IL-4 or the paracrine interaction between macrophages and cancer in the context of a tumour microenvironment.

## Materials And Methods

### Materials

Coomassie blue (Coomassie Brilliant Blue R-250) was from Bio-Rad (Gladesville, NSW, Australia). Cell culture media, serum and supplements were from Life Technologies, Mulgrave, VIC, Australia. Morphine and naloxone were from Hospira (Mulgrave, VIC, Australia). Methylnaltrexone was from Link Medical Products (Warriewood, NSW, Australia). Unless otherwise stated, all other reagents were from Sigma-Aldrich (Castle Hill, NSW, Australia).

### Cell culture

Mouse RAW264.7 macrophages were maintained in Dulbecco’s modified Eagle’s medium (DMEM) supplemented with 10% (v/v) foetal bovine serum (FBS), penicillin (100 units/ml) and streptomycin (100 μg/ml). Murine mammary breast carcinoma cells (4T1) were maintained in Roswell Park Memorial Institute medium (RPMI-1640) containing 5% FBS (v/v), 1% sodium pyruvate (v/v), penicillin (100 units/ml) and streptomycin (100 μg/ml). Bone marrow (BM) was isolated from femurs of 12 week-old C57BL/6l mice following procedures approved by the University of Queensland Animal Welfare Unit Ethics Committee (571/12). Bone marrow-derived macrophages (BMDM) were generated by culture of BM for 6 days in DMEM containing 20% L929 cell-conditioned media, 10% heat-inactivated foetal bovine serum, 1mM sodium pyruvate, 2 mM L-glutamine and 10 mM HEPES. Fresh media was added on days 2 and 4 of culture. All cells were incubated in a humidified atmosphere at 37^o^ C with 5% CO_2_.

### Polarization of macrophages

RAW264.7 or BMM cells were seeded at 2 × 10^5^ cells per well in a 12 well plate with 1 ml of serum-containing DMEM and incubated overnight. The cells were incubated with serum-free medium containing 0–20 ng/ml recombinant murine IL-4 (PeproTech, Rocky Hill, NJ, USA) to induce alternatively activated macrophage (M2) polarization. After 24 h (BMM) or 48 h (RAW264.7) incubation, conditioned media and cells were collected for gelatine zymography and real-time reverse transcriptase polymerase chain reaction (Real time RT-PCR), respectively.

### Co-cultures

Mouse macrophages and 4T1 cells were seeded individually or together in equal numbers (1 × 10^5^ cells) in a 12-well plate Transwell® system with a porous polycarbonate membrane filter (0.4  μM pore size, Corning, NSW, Australia) in a 1:1 mix of complete medium of each cell line for 18 h. The medium was replaced with a 1:1 mix of serum-free RPMI and DMEM media, and cells incubated with 10–20  μM DBL® morphine sulfate (Mulgrave, VIC, Australia). The 48 h (RAW264.7 and 4T1) or 24 h (BMM) conditioned media and cells were collected, tested for gelatine zymography and real time RT-PCR, respectively.

### Gelatine zymography

Conditioned media were analysed for gelatinases (MMP-2 and MMP-9) quantification level using gelatine zymography as described previously^8^. Briefly, equal protein amount (as measured using Bio-Rad DC^TM^ Protein assay) of the conditioned media were loaded on an 11% polyacrylamide gel containing 1% (w/v) gelatine and separated electrophoretically in non-reducing conditions. The gels were placed in a renaturing solution containing 5 mM CaCl_2_, 50 mM Tris and 2.5% (v/v) Triton X-100 overnight. The gel was then incubated in a solution of 5 mM CaCl_2_ and 50 mM Tris–HCl at 35 °C for 3 h to allow enzyme activity. After Coomassie staining (45% (v/v) methanol, 10% (v/v) glacial acetic acid and 0.25% (w/v) Coomassie Blue R-250) the gel was destained with a solution containing 10% (v/v) acetic acid and 25% (v/v) methanol until areas containing the proteolytic enzymes appeared as clear bands on a dark background. Band intensity was determined following high-resolution flatbed scanning and image analysis using Image J software.

### Invasion assay

24 h prior to the assay, RAW264.7 cells (2 × 10^5^ cells) were seeded in the bottom chamber of a 24-well plate. Moreover, 4T1 cells were incubated in serum-free RPMI medium. 4T1 cells were harvested and 1 × 10^5^ cells were added to the upper chamber of a BME coated polycarbonate (PC) membrane filter chamber (8 μM pore size, BioScientific Pty Ltd, Sydney, N.S.W.). The medium was replaced with a 1:1 mix of serum-free RPMI and DMEM media. Morphine sulfate (DBL® Mulgrave, VIC, Australia) was added to upper and bottom chamber at a concentration of 20 μM. Invasion was allowed to proceed for 48 h. A negative control was set up where 4T1 cells were seeded in the upper chamber with no RAW264.7 in the bottom well. After incubation the inserts were washed with PBS twice and fixed with buffered formalin (ALPHELYS, Plaisir, France) for 20 min. The membranes then were stained overnight using crystal violet (Sigma-Aldrich, Castle Hill, NSW, Australia). Inserts were imaged in their centre at 20X magnification and cells per field were quantified in three independent experiments.

### Quantitative RT-PCR

Total RNA derived from RAW264.7 and 4T1 cells was isolated and purified using the RNeasy Plus Mini Kit (Qiagen, VIC, Australia). RNA (600–2000 ng) was reverse transcribed using the Omniscript RT Kit (Qiagen, VIC, Australia) and amplified using TaqMan® Fast Universal PCR Master Mix (Life Technologies, VIC, Australia) with AmpliTaq Gold® DNA Polymerase, and TaqMan® Gene Expression Assays including Arginase-1 (Mm00475988_m1), MMP-9 (Mm00442991_m1) and MRC-1 (Mm00485148_m1) in a StepOnePlus 7500 real time PCR system (Applied Biosystems, Carlsbad, CA, USA). Quantification was performed relative to 18S ribosomal RNA using the comparative critical threshold (Ct) method^31^ and relative expression of the target gene measured in at least three separate experiments was compared with the appropriate control.

### Statistical analysis

Data are presented as mean values ± S.E.M and were analysed, using Graphpad Prism software (version 5.04). Differences between single group means with control means were analysed using one tailed Student’s *t*-test and one-way or two-way analysis of variance (ANOVA) where appropriate. Differences were considered statistically significant at p < 0.05.

## Additional Information

**How to cite this article**: Khabbazi, S. *et al.* Morphine Modulates Interleukin-4- or Breast Cancer Cell-induced Pro-metastatic Activation of Macrophages. *Sci. Rep.* **5**, 11389; doi: 10.1038/srep11389 (2015).
